# Exploring Cotard’s Delusion Within the Context of Major Depressive Disorder With Psychotic Features: A Case Report

**DOI:** 10.7759/cureus.68047

**Published:** 2024-08-28

**Authors:** Alina F Faunce, William B Tennant

**Affiliations:** 1 Research, Alabama College of Osteopathic Medicine, Dothan, USA; 2 Psychiatry, Turning Point Psychiatry, Cullman Regional Medical Center, Cullman, USA

**Keywords:** nihilistic delusions, venlafaxine, biopsychosocial model, cotard's delusion, unipolar psychotic depression

## Abstract

Cotard’s delusion is a rare and complex condition marked by profound detachment from reality and nihilistic beliefs about decay and mortality, often stemming from underlying psychiatric or neurological issues. In the case of Mr. B, a 44-year-old man with a history of seizure disorder and major depressive disorder (MDD) with psychotic features, his delusions included beliefs that his right leg was decaying and that he was deceased. Despite treatment attempts with various combinations of antidepressants and antipsychotics, his delusions worsened, achieving substantial relief only through venlafaxine monotherapy. This outcome, highlighting the potential inefficacy of antipsychotics and the success of venlafaxine, underscores the need for better understanding and additional pharmacological research into Cotard’s delusion, particularly within the context of MDD with psychotic features. The exact mechanism through which venlafaxine achieved a positive response remains unknown, necessitating further comprehensive studies.

## Introduction

Cotard's delusion manifests as a complex condition marked by a profound detachment from reality or existence itself. It is entwined with nihilistic convictions centered on decay, mortality, and loss of blood or internal organs. A French psychiatrist and neurologist first described it in 1880 as *délire des négations*, later naming it Cotard's delusion [1]. Cotard's delusion is believed to be a symptom of an underlying neuropsychiatric condition rather than a distinct disorder, which is why it is not classified separately in the Diagnostic and Statistical Manual of Mental Disorders 5th edition (DSM-5). Delusional disorder, first introduced in 1883, is recognized in the DSM-5 and includes various subtypes such as erotomanic, persecutory, somatic, grandiose, bizarre, or unspecified [2]. Delusional misidentification syndromes (DMS), categorized in 1986, are a group of disorders involving the belief that the identity of a person, place, or thing has been changed or altered. DMS encompasses disorders such as Capgras syndrome, the delusion that an identical imposter has replaced a close family member or friend, discovered in 1923, and Reverse Capgras syndrome, where the person believes an imposter replaces themselves. In 1927, Fregoli syndrome was identified; this disorder is characterized by the delusional belief that different people are the same person, but that person constantly changes their appearance. Cotard's delusion is sometimes considered part of the delusional misidentification syndrome group [3].

Clinically, this rare disorder arises alongside psychiatric or neurological disorders, and both factors need to be considered. On the psychiatric side, Cotard's delusion is associated with postpartum depression, depersonalization disorder, catatonia, and mental retardation. On the neurological side, it is associated with frontotemporal atrophy, epilepsy, brain tumors, encephalitis, traumatic brain injury, multiple sclerosis (MS), and cerebral infarctions [4]. It is most commonly seen in middle-aged adults with a history of chronic mood disorders and is rarely reported in childhood or adolescence [5]. Cotard's delusion manifests in 0.11% of neurological patients and 0.62% of psychiatric patients [6]. Due to its rarity and potential underdiagnosis, most literature consists of case studies, leading to difficulty in deciding the best clinical guidance to offer patients suffering from Cotard's delusion [7].

This case report examines the atypical presentation of Cotard's delusion in unipolar psychotic depression, detailing 'Mr. B's' journey through various unsuccessful combination therapies. Mr. B's presentation of Cotard's delusion is atypical in the setting of unipolar psychotic depression, given that this delusion is more commonly associated with schizophrenia, major depressive disorder (MDD), and bipolar disorder in a psychiatric presentation. His delusions centered on cenesthopathic beliefs that his right leg was undergoing decay and would eventually detach, alongside a perception of an alternate version of himself inhabiting his leg. Additionally, he experienced pervasive sensations of having died, being deceased, or facing imminent demise.

Through thorough history and examination, Mr. B's delusion was discovered as a symptom of underlying unipolar psychotic depression. According to current American Psychiatric Association (APA) guidelines, the recommended treatment approach for unipolar psychotic depression is a combination therapy of an antidepressant and an antipsychotic, electroconvulsive therapy or lithium [8]. Despite exacerbation with the recommended antipsychotics for his diagnosis, Mr. B achieved mood and delusion relief through venlafaxine monotherapy. He likewise experienced decreased delusion frequency with the addition of lurasidone, an unusual antipsychotic for the treatment of unipolar psychotic depression. This highlights the necessity for an enhanced understanding of the biopsychosocial approach and pharmacological exploration of Cotard's delusion beyond its traditional association with schizophrenia, emphasizing the need for further research to elucidate effective therapeutic mechanisms.

## Case presentation

'Mr. B,' a 43-year-old male, presented to this clinic via referral from his primary care physician. His medical history includes seizure disorder, obstructive sleep apnea (OSA), MDD, insomnia, generalized anxiety disorder (GAD), and type 2 diabetes mellitus managed with insulin. He says the relationship with his wife and two children is good and okay. He visits a neurologist every 3-6 months since his first seizure in 2000, which he attributes to a heat stroke; his last seizure was reportedly in 2014. He recalls his neurologist saying something about scar tissue on the right frontoparietal region but doesn't know anymore about this and was diagnosed with an "anxiety-related seizure disorder." He also has a past medical history of an abnormal EKG of wolf-parkin-white, which was corrected with ablation in 2016, and has had normal EKGs since. He has no history of prior psychiatric hospitalizations, substance abuse, previous suicide attempts, or self-harm. 

On Mr. B's first visit in February 2022, he expressed concern about increased guilt, decreased energy, anhedonia, and difficulty falling asleep due to ruminating thoughts about death while displaying psychomotor agitation in the form of fidgeting. He reported auditory hallucinations, including the television talking to him and conversations with his six-year-old son that he knows are not real. He cannot elaborate on the details but states it is mildly distressing. He states that this occurs less than once a week. Mr. B denies any stressors or traumas that may have led to any of his symptoms. He denied any suicidal ideation, homicidal ideation, or auditory or visual hallucinations at this time. Based on his mood-congruent delusions and hallucinations that only occur alongside a major depressive episode, he was diagnosed with MDD with psychotic features or unipolar psychotic depression. 

At that time, he was only taking levetiracetam XR 500mg BID for seizure disorder prescribed by his neurologist. He had previously failed several psychiatric medication trials (escitalopram, aripiprazole, sertraline, paroxetine, and mirtazapine) aimed at addressing his MDD. These were all eventually discontinued due to unsatisfactory remission, adverse side effects (most notably fatigue or lethargy), or increased delusion frequency. He cannot elaborate on how long he took each of these medications, who prescribed them, or when he trialed them. At this visit, the plan was to continue watchful monitoring for remitting seizures to postictal psychosis from his history of seizures, untreated sleep apnea, insomnia, and psychosis.

Throughout 13 follow-up visits spanning from February 2022 to April 2024, Mr. B's symptoms evolved, revealing a complex diagnostic picture of psychotic nihilistic delusions and unusual spells later attributed to functional neurologic symptom disorder (FNSD).

In his initial sessions, he experienced night terrors where he would sit up in bed screaming about imminent death, occurring most frequently in the hypnopompic/hypnogogic period. He reported overwhelming emotions of impending doom and heard children saying things that "they aren't saying" but does not remember what they say. Venlafaxine 75mg QD was initiated, which helped improve his mood, although occasional ideas of reference persisted. Additionally, he reported feelings of arms or legs falling off; Mr. B continued to experience distressing delusions and episodes where he felt his right leg was decaying and there was another version of himself in his right arm or leg. Mr. B also noted that topiramate 200mg BID was added to his regimen by his neurologist, but he does not remember why. Brexpiprazole 1mg QD was then added to address his persistent delusions and hallucinations, later increased to 3mg QD due to persistent symptoms. However, this led to increased anxiety and panic attacks, prompting a return to venlafaxine monotherapy.

Mr. B was educated on potential links between his mood, thoughts, and behavior due to levetiracetam; he was recommended to discuss this with his neurologist. Lithium was briefly added alongside venlafaxine but discontinued due to upper extremity tremors and its lack of efficacy for mood symptoms, although he denied any hallucinations. Mr. B continued venlafaxine 75mg QD, with plans for ongoing collaboration with his neurologist. Mr. B also reported episodes where he believes his wife and sons are uninterested in his well-being, which worries his children.

Over time, venlafaxine was titrated up to 225mg QD, significantly improving his mood. Family therapy was encouraged to address ongoing conflicts with his wife, although he noted her reluctance to participate. Despite the progress, Mr. B continued to experience occasional episodes of feeling dead, thoughts of imminent death, and struggles in his relationships. He reports good sleep but mentions that he and his wife no longer share a bed, and she blames him for everything, making him feel unsupported. Despite improvements in his mood symptoms, he still experiences "low" moments, which he typically attributes to issues in his marriage. He felt particularly low because his wife forgot his birthday and did not inform their children, which has been bothering him for a few weeks. He expresses frustration that she remains unsupportive of him and his therapy.

At his most recent visit, he reported experiencing episodes of freezing, where he would black out and talk in gibberish or scream, eventually attributed to FNSD spells, as confirmed and diagnosed by his neurologist. Those with neurological conditions such as epilepsy or MS can present with contaminant conversion (FNSD) disorder. This diagnosis explains his symptoms of slurred speech, paralysis, and nonepileptic seizures. These episodes have intensified marital and family tensions. Learning of these episodes, his wife forbade him from picking up their children. He also described a sensation of having another person on his shoulder and feeling dead in his arm, although these symptoms have occurred only a few times. Venlafaxine 225mg QD was continued, and a trial of lurasidone 40mg QHS was initiated to address recurring delusions. Following a two-month lurasidone trial, Mr. B reported no psychotic episodes or delusions during this time. He mentioned that his mood has improved with lurasidone, although he still feels his spouse is unsupportive of his pursuit of therapy. He continues to report some FNSD episodes, and regular appointments with his neurologist have been scheduled to manage this diagnosis.

## Discussion

Mr. B's case presents multi-faceted diagnostic complexity due to the evolving nature of symptoms and the overlap between his psychiatric and neurological conditions. Distinguishing between symptoms of psychotic depression, GAD, and FNSD requires careful assessment and ongoing monitoring. Finding the right combination of medications to effectively manage Mr. B's symptoms while minimizing side effects is challenging for the same reason. Numerous past psychiatric medication trials have resulted in various adverse effects, highlighting the need for close monitoring and adjustment of his medication regimen. Additionally, staying motivated and engaged in the therapeutic process can be challenging, especially when dealing with long-term or complex issues. Coordinating with other professionals can present communication and treatment planning challenges when multidisciplinary care is necessary. Accurate and timely assessment should expedite treatment and avoid unnecessary medical interventions. Creating a supportive environment and a collaborative treatment relationship that improves Mr. B's' understanding of his symptoms have been shown to help those diagnosed with FNSD engage in appropriate treatments [9].

Addressing Mr. B's psychosocial stressors is crucial for improving his overall well-being [10]. Mr. B, previously an engineer, lost his job due to interference from FNSD spells at the job site. None of these episodes described were psychotic or reached a level of needing inpatient admission. He subsequently had to file a Social Security disability claim due to his FNSD spells, which caused further tension with his wife. The combination of his strained relationship with his wife, job loss, and inability to provide for his family led to feelings of guilt, inferiority, and ineptitude. In cases of psychotic depression, the psychotic features often revolve around intense guilt and feelings of worthlessness [11]. Encouraging family therapy and providing support for coping with interpersonal challenges would enhance pharmacological treatment [12].

Combination therapy with an antipsychotic and antidepressant, antipsychotics, and mood stabilizers are frequently used to treat delusional disorder, as well as psychotherapy and cognitive behavioral therapy as a means of treatment. However, further large-scale research is needed to effectively in the treatment of delusional disorders, so treatment includes those that are considered adequate for each patient. Antidepressant monotherapy with venlafaxine was found greatly helpful to Mr. B with his mood and delusions. This is particularly unique due to the typical precaution in prescribing antidepressant monotherapy due to triggering a psychotic episode. Additionally, lurasidone is not typically an adjunct medication for MDD or MDD with psychotic features, and this has been helping Mr. B's symptoms as well. In these cases, a second-generation antipsychotic with a more favorable side effect profile is generally used. For example, aripiprazole can be started at 2 to 5 mg daily and gradually increased over several days or weeks to 10 to 20 mg daily while monitoring for clinical response. Once a therapeutic dose is achieved, we allow one to two weeks at that dose before evaluating the effect and need for further dose increase or medication switch. For patients who have difficulty with adherence, long-acting injectable antipsychotic formulations are a reasonable alternative to oral medications [8]. Clozapine, a second-generation antipsychotic indicated for treatment-resistant psychosis, may be an appropriate alternative intervention when the patient has depression and is a high suicide risk. 

Mr. B's case exemplifies the biopsychosocial model by considering the intricate interplay of biological, psychological, and social factors in understanding and treating his complex psychiatric and neurological presentation. Mr. B's symptoms, such as auditory hallucinations, somatic delusions, and neurological manifestations, highlight the biological underpinnings of his condition. Pharmacotherapy, including antidepressants, antipsychotics, and antiepileptic drugs, targets these biological aspects to alleviate symptoms. Furthermore, his history of seizures and the subsequent diagnosis of FNSD underscore the importance of considering neurological factors in his treatment. Mr. B's psychiatric symptoms, such as mood fluctuations, delusions, hallucinations, and cognitive distortions, reflect underlying psychological processes. Interventions such as cognitive-behavioral therapy complement pharmacotherapy in addressing these factors, highlighting the importance of holistic treatment approaches.

Additionally, Mr. B's distressing experiences, such as his marital issues and feelings of isolation, highlight the psychological stressors contributing to his condition. The psychosocial implications of Mr. B's case are evident in the significant role of social dynamics in his presentation and treatment. Discord within his family and challenges in interpersonal relationships contribute to his distress and complicate his recovery process. Expressed emotion from family or caregivers is shown to have a direct association with the recurrence of psychiatric illness [13]. Family therapy is recommended to address these social stressors and improve his overall well-being.

Navigating Mr. B's complex medical and psychiatric conditions, along with their interplay, introduces significant intricacy to his treatment plan. Balancing the administration of psychiatric medications and antiepileptic drugs necessitates close collaboration between psychiatric and neurology specialists. Insight from neurology revealed a history of levetiracetam use, which may have impacted his mood symptoms [14]. Despite adjustments to his antiepileptic medications, Mr. B's neurological symptoms persisted, ultimately leading to a diagnosis of FNSD.

Improvement in early insomnia has been linked to positive outcomes in the pharmacotherapy of psychotic depression, irrespective of the medication used [15]. Therefore, managing Mr. B’s insomnia and OSA could potentially improve his psychotic depression. There is an increased prevalence of OSA in individuals with MDD and PTSD. This is hypothesized to be linked to dysregulation of the hypothalamic-pituitary-adrenal axis, but more research needs to be done to gather evidence for other psychiatric disorders, mood disorders, and anxiety disorders [16].

However, Mr. B’s reluctance to use his CPAP machine presents another barrier to achieving his full mental health potential. The most significant barrier to treating his psychotic depression remains his strenuous family dynamics. Addressing these through various family interventions has shown improved outcomes in multiple psychiatric illnesses. In Mr. B's case, family psychoeducation effectively addresses many of his stressors. It would reduce expressed emotion and criticism directed toward him, coordinate shared goals with the family, support the patient, understand family members’ expectations, and engage families as equal partners in treatment planning and delivery [17]. Among patients with major depression, family therapy has been shown to result in greater improvement and reduced suicidality compared to treatment without family therapy. Couples counseling, in addition to individual therapy, would be beneficial in his case.

The APA designated behavioral activation as having empirical solid support for the treatment of depression and commitment therapy as having a modest level of empirical support for psychotic depression. These two therapies share that experiential avoidance, or the attempt to escape unwanted thoughts and feelings, is essential in the development and maintenance of psychopathology [18]. Experiential avoidance is additionally related to hallucinations and predicted depression and anxiety symptoms [19,20]. These interventions could improve Mr. B’s depressive and psychotic symptoms and overall well-being.

## Conclusions

Mr. B's case highlights the need for a comprehensive multidisciplinary approach when managing complex comorbid psychiatric and neurologic disorders. Addressing pharmacological and psychosocial factors and providing individualized care are essential for effective treatment. Particular focus should also be given to family involvement in Mr. B's treatment. While combination therapy shows promise, further pharmacological research is still needed to manage unipolar psychotic depression and the symptoms of delusional misidentification syndromes like Cotard's syndrome. Supportive interpersonal relationships can improve patient outcomes, and a holistic, collaborative approach is essential for optimizing care and enhancing the quality of life.
